# Young People’s Satisfaction With the Online Mental Health Service eheadspace: Development and Implementation of a Service Satisfaction Measure

**DOI:** 10.2196/12169

**Published:** 2019-04-17

**Authors:** Debra Rickwood, Alison Wallace, Vanessa Kennedy, Shaunagh O’Sullivan, Nic Telford, Steven Leicester

**Affiliations:** 1 headspace National Youth Mental Health Foundation Melbourne Australia; 2 University of Canberra Canberra Australia

**Keywords:** mental health, adolescent, telemedicine, counseling, internet, satisfaction, feedback, telehealth, young people

## Abstract

**Background:**

Online youth mental health services are an expanding approach to meeting service need and can be used as the first step in a stepped-care approach. However, limited evidence exists regarding satisfaction with online services, and there is no standardized service satisfaction measure.

**Objective:**

This study implemented an online youth mental health service satisfaction questionnaire within eheadspace, an online youth mental health service. The aims were to test the questionnaire’s psychometric properties and identify current levels of satisfaction among service users, as well as to identify client and service contact characteristics that affect satisfaction.

**Methods:**

Data were collected from 2280 eheadspace clients via an online questionnaire advertised and accessed through the eheadspace service platform between September 2016 and February 2018. Client and service contact characteristics, potential outcomes, and session and service feedback data were collected.

**Results:**

The service satisfaction questionnaire demonstrated high internal consistency for the overall satisfaction scale (alpha=.95) and its three subscales: session satisfaction, potential outcomes, and service satisfaction. A three-factor model was the best fit to the data, although including a higher order unidimensional construct of overall satisfaction was also a reasonable fit. Overall, young people were very satisfied with eheadspace (mean 3.60, SD 0.83). Service characteristics, but not client characteristics, were significantly associated with satisfaction. Young people were more satisfied with eheadspace when they had greater engagement as evident through receiving esupport rather than briefer service provision, having a longer session and greater interaction with the clinician, and not previously attending a face-to-face headspace center.

**Conclusions:**

The online youth mental health service satisfaction questionnaire developed for and implemented in eheadspace showed good psychometric properties. The measure is brief, has good internal consistency, and has a clear factor structure. The measure could be adapted for use in other online youth mental health services. The young people using eheadspace and completing the feedback survey were highly satisfied. Greater engagement with the online service was shown to be associated with greater satisfaction. No specific client demographic groups were shown to be more or less satisfied.

## Introduction

Mental health problems peak in young people between the ages of 12 and 25 years [1], and more than one in four young people experience a mental illness each year [2]. Three-quarters of all mental health problems have their first onset before the age of 25 [3]. The delay between first symptoms and access to effective treatment can extend to 15 years [4]. Poor mental health in childhood and adolescence increases vulnerability to mental health problems in adulthood, revealing the importance of prevention and early intervention for this age group [5-7]. It has been estimated that up to half of lifetime mental illnesses may be prevented through early intervention and prevention methods [8]. Yet, only 13% of young men and 31% of young women seek professional help [9]. Although treatment rates have improved in Australia following the introduction of subsidized services, help-seeking remains substantially lower for mental health problems compared to physical health conditions [10].

Online mental health services have emerged as an innovative way to promote self-referral, increase help-seeking behaviors, and meet the high level of unmet service demand, through accessible youth-friendly technology [11,12]. The internet is the main source of information for young people [13], and the Australian Bureau of Statistics reports that 98% of youth aged 15 to 17 years, and 97% of young adults aged 18 to 24 years, accessed the internet during 2014 to 2015 [14]. The internet has escalated as a source of information for mental health issues in recent years [15-17]. A cross-sectional study of 2000 Australian young people aged 12 to 25 years in 2008 found that 30.8% seek online mental health information and that 21% of those aged 12 to 17 years and 34% of those aged 18 to 25 years reported searching online for mental health help [18]. Factors associated with internet use for mental health information included being female and using the internet after 11 pm. Research also shows that young people feel comfortable accessing online information about mental health issues, which may improve help-seeking behaviors for those who may not otherwise seek help using traditional approaches [19].

A growing range of online options are available, from self-help psychoeducation to interactive sessions with a mental health clinician [11]. These can be incorporated within integrated systems of mental health care, including emerging stepped-care approaches, which are staged systems comprising a hierarchy of interventions from least to most intensive that are matched to an individual’s needs [20]. Although such approaches need to be flexibly implemented so that they are appropriate to severity, need, and stage of presentation, online resources and services can comprise part of the spectrum of interventions [21]. Importantly, e-therapy can be included in ways that complement and extend traditional therapeutic approaches. E-therapy has been shown to be particularly effective for mild to moderate presentations and for the common mental health problems of anxiety and depression, but applications in more complex and serious presentations are also emerging [22]. Online services have many advantages, including increasing mental health literacy [23], potentially improving help-seeking behaviors for face-to-face therapy [4], and providing support that is easily and widely accessible, while reducing the costs to service providers as part of an integrated system of care [24].

Nevertheless, the growth of mental health services available online provides a challenge for young people, because there is a wide range of choice that is difficult to navigate [11]. Importantly, most young people self-refer to online services [25], so it is important that they are directed to high-quality and evidence-based services [26]. Service satisfaction feedback to measure young people’s experiences, the quality of services, and outcomes achieved is essential for service development, and to ensure that young people can access online services that meet their needs.

Service satisfaction is a broad term that can encompass a range of factors relating to care. Self-report feedback tools provide service providers with important information regarding clients’ progress [27]. Regular feedback is recommended for all mental health practice, as clinicians are not always able to recognize treatment failure as it is happening [28,29]. However, there is a lack of formal feedback measures for child therapy, giving young clients “little voice in the services they receive” [30]. Simmons et al [31] emphasized the importance of satisfaction scales covering the relevant areas for youth mental health because much research to date in youth satisfaction has focused on parental or caregiver satisfaction [32].

The few examples of youth-targeted service satisfaction measures include the Multidimensional Adolescent Satisfaction Scale [33] and the headspace Youth (mental health) Service Satisfaction Scale [31,34]. The Youth Service Satisfaction Scale is one of the few service satisfaction measures specifically developed for use in early intervention youth mental health services for young people aged 12 to 25 years. It has demonstrated good psychometric properties and revealed a high level of satisfaction with headspace center-based (face-to-face) services. To our knowledge no youth satisfaction questionnaires targeted toward online services exist; therefore, this study aimed to redress this gap.

This study involved the development and implementation of a service satisfaction questionnaire in an online youth mental health service—eheadspace—an online mental health support and counseling service for young Australians aged 12 to 25 years implemented as part of headspace, the National Youth Mental Health Foundation funded by the Australian Government’s Department of Health. The eheadspace service provides free and confidential access to mental health clinicians via Web chat, email, and phone from 9 am to 1 am AEST 7 days per week. This extends the reach of headspace centers, which provide face-to-face services through 110 centers across Australia. Like headspace centers, young people can access eheadspace for any reason, although the service is promoted as providing support and counseling. The eheadspace service began in 2011, in recognition of the high demand for youth mental health care that could not be met by face-to-face services alone. There has been a high level of uptake of the eheadspace service, and young people have been shown to access the service when they are feeling highly distressed [25]. However, many young people only access once, and there has been little examination to date of young people’s experience of and their satisfaction with this service approach. Understanding user satisfaction is essential to avoid premature disengagement and to ensure that young people receive a service that they value and that meets their needs.

The aims of this study were to:

1. Adapt the headspace center Youth Service Satisfaction Scale for use in eheadspace and examine its psychometric properties;
2. Establish current levels of satisfaction among young people accessing eheadspace; and
3. Determine whether satisfaction varies according to client characteristics or characteristics associated with the service contact.

## Methods

### Participants

Participants consisted of 2280 young people aged 12 to 25 years (mean 17.8, SD 3.2), who registered with eheadspace Web chat between September 20, 2016 and February 20, 2018, wanted help for themselves (rather than a friend), and completed at least one service satisfaction survey. Young people were included if they received a direct eheadspace service, which could be either (1) a brief service provision engaging the young person, identifying the presenting problems, and using brief therapeutic strategies to address these problems; or (2) an esupport-targeted strategy delivered following initial contact and engagement of the young person guided by a care plan and following a full assessment.

All young people registering for eheadspace Web chat during the survey period were invited to voluntarily participate. The survey response rate was 8.7%, from a total of 26,234 eligible young people who accessed the service during this period. The eheadspace users can complete a feedback survey each time they access the service; however, if users had completed more than one survey, only the first complete survey was analyzed in this paper.

### Procedure

Service satisfaction surveys were collected from eheadspace clients via an online questionnaire advertised and accessed through the eheadspace service platform. The platform provides other service options (eg, a link to the Digital Work and Study Service and support for family and friends), but only young people who used the eheadspace online service were offered the service satisfaction questionnaire. To access eheadspace online, a user clicks on a link to “online chat” and then provides required registration or log-in information. When a young person logged in and selected the online chat option, a blue box appeared on the side, asking: “Do you have a spare minute or two to complete our survey about your chat today? Thanks in advance if you do.” Young people were invited to click a link to access the questionnaire once they had finished their online chat, which subsequently directed them to the participant information page. Participation was voluntary.

Survey data were combined with demographic information, clinical characteristics, and service information that was extracted from the eheadspace administrative Minimum Data Set. All young people electronically enter data when they first log in to access the service, and each time they access the service and it has been 14 or more days since they last did so. All clinicians enter data at the end of each service session.

Ethics approval was obtained from Melbourne Health.

### Measures

#### Client Characteristics

Demographic characteristics included gender (male, female, trans, intersex, and another gender [please specify]), age group (12-17 or 18-25 years), sexuality (heterosexual, lesbian, gay, bisexual, questioning, and other sexuality [LGBTIQ]), Aboriginal and/or Torres Strait Islander person (yes or no), and area of residence (metropolitan or regional). Note that gender was analyzed only as male or female due to the small sample size for the nonbinary categories.

Reason for attending was measured using clinician-rated responses to a list of options asking the main issue for which the young person presented. This was categorized into mental health or other issues. Other issues included situational issues, vocational assistance, alcohol or other drugs, physical health, sexual health and “other.”

Clinical stage was estimated for those presenting with mental health issues to provide a general indication of diagnostic complexity. Clinicians were asked to estimate an approximation of the clinical stage using responses from options based on the clinical staging heuristic [35]. Stage 0 indicated none or few depressive or anxiety symptoms and little or no impairment in functioning; stage 1a indicated mild to moderate *Diagnostic and Statistical Manual of Mental Disorders-IV* (DSM-IV) disorder of depression, generalized anxiety disorder (GAD), or impairment in functioning; stage 1b indicated stage 1a plus additional features falling short of stage 2; stage 2 indicated moderate to severe symptoms meeting threshold diagnosis; stage 3 indicated periods of remission (<6 months of symptom improvement) but no recovery; and stage 4 indicated ongoing severe symptoms with no asymptomatic periods. If the young person accessed eheadspace for a non-mental health-related issue, “not applicable” was selected; if the clinician was unable to adequately assess clinical stage, “not enough information available” was selected.

#### Service Contact Characteristics

Service characteristics included the type of direct service (brief service provision or esupport), wait time before an eheadspace session (the time between the user completing their registration and log-in for the online chat and the time the clinician responds, ranging from 0 to 106 minutes), session start time (9 am to 1 am), session duration (<15 minutes to ≥90 minutes), number of words conveyed by the young person (0 to 2599 words), number of words conveyed by the clinician (0 to 3583 words), and the number of times the young person had previously received a direct service (0 to 2 or more sessions). Young people were also asked if they had visited a headspace center prior to using eheadspace (yes or no) and referral information (self-referred or referred by other).

### Service Satisfaction

To develop a measure of eheadspace service satisfaction, relevant items were adapted from the headspace center Youth Service Satisfaction Scale [34] and supplemented by items from a review of existing satisfaction surveys and consultation with eheadspace staff and clients. This resulted in 13 items comprising statements that were each responded to on a 5-point Likert scale from strongly disagree to strongly agree, with higher scores indicating higher levels of satisfaction. The items focused on three different satisfaction domains:

1. Session satisfaction: session focused on young person’s main concern, young person felt listened to and understood, session helped the young person to understand their situation more clearly, young person was provided with skills or resources to help them manage their situation going forward, session made young person feel more hopeful and optimistic.
2. Potential outcomes: young person feels better day-to-day, can manage the things they do better, copes better, has improved relationships.
3. Service satisfaction: young person feels comfortable sharing information with eheadspace clinicians, eheadspace is easy to use, young person would recommend eheadspace to a friend, eheadspace met the young person’s expectations.

### Analysis

Data were analyzed using IBM SPSS version 24 and AMOS version 25. An exploratory principal components factor analysis of the 13 original eheadspace feedback survey items was first conducted. Confirmatory factor analysis (CFA) with maximum-likelihood estimation was then used to further test the factor structure. Cronbach reliability analysis was conducted to confirm internal consistency.

Descriptive statistics were computed for all variables. Independent *t* tests and one-way analyses of variance were then used to determine group differences in satisfaction scores based on client characteristics (gender, age group, sexuality, Aboriginal and/or Torres Strait Islander status, areas of residence, reason for attending, clinical stage), and service contact characteristics (type of service, wait time before session, session start time, duration, prior access to a headspace center, number of previous direct service sessions, word count per client and clinician, referral information). Significance was set at *P*<.01 due to the high power from the large sample size.

## Results

### Client and Service Contact Characteristics

Of the 2280 participants, 77.76% (n=1773) were female, and 55.04% (n=1255) were aged between 12 and 17 years, whereas 44.96% (n=1025) were between 18 and 25 years. There were 64.96% (1481/2280) who identified as heterosexual, 70.44% (1606/2280) who lived in a metropolitan area, and 3.77% (86/2280) who identified as an Aboriginal and/or Torres Strait Islander person.

For presenting issue, 58.82% (1341/2280) contacted eheadspace for mental health problems and 26.32% (600/2280) contacted for another issue (14.87%, 339/2280 missing). Of those with a clinical stage rating (ie, excluding “not applicable” and “not enough information available”), more than half (54.38%, 522/960) were rated stage 1a indicating mild to moderate DSM-IV disorder of depression, GAD, or impairment in functioning.

The majority of clients (85.13%, 1941/2280) had an esupport session, whereas 14.87% (339/2280) had a brief service provision session on the day of completing the survey. Half (50.39%, 1149/2280) the sessions lasted 31 to 60 minutes, but sessions ranged from less than 15 minutes to 90 or more minutes. More than half (57.96%, 1125/1941) of esupport sessions lasted 31 to 60 minutes, whereas only 7.08% (24/339) of brief service provisions lasted this long. Only 5.04% (115/2280) did not have to wait to access eheadspace when they visited, 41.49% (946/2280) had to wait less than 5 minutes, and 53.46% (1219/2280) had to wait more than 5 minutes (maximum wait time was 106 minutes). Most (83.29%, 1899/2280) sessions occurred before 11 pm, but 16.71% occurred between 11 pm to 1 am. On average, young people inputted a mean 397.01 (SD 290.05) words, compared to a mean 686.18 (SD 343.25) words from clinicians. The majority of young people (71.84%, 1638/2280) input fewer than 500 words per session, compared with only 30.04% of clinicians (685/2280). Most young people (67.72%, 1544/2280) self-referred to eheadspace, with 30.35% (692/2280) referred by others. Only 21.80% (497/2280) of participants had visited a headspace center prior to accessing eheadspace. Before the session on the day of survey completion, 64.04% (1460/2280) had not previously received esupport, 16.49% (376/2280) had previously received one, 7.54% (172/2280) had previously received two, and 11.84% (270/2280) had previously received three or more esupport sessions.

### Factor Analysis of Satisfaction Scale

The 13 original items of the eheadspace feedback survey were subjected to principal components analysis (PCA) to identify the factorial structure. Inspection of the correlation matrix revealed all coefficients above 0.30. The Kaiser-Meyer-Olkin value observed was 0.95, which exceeded the recommended value of 0.60 [36], and the Bartlett test of sphericity [37] was statistically significant, supporting the factorability of the correlation matrix.

The PCA revealed the presence of one component with an eigenvalue of 8.3, explaining 63.8% of the variance. Standardized factor loadings for 12 of 13 items were strong and positive (range 0.64-0.87), except for “eheadspace is easy to use,” which was 0.50. The eigenvalues and factor loadings supported a unidimensional solution, and an inspection of the scree plot revealed a clear break after the first factor [38].

**Figure 1:**
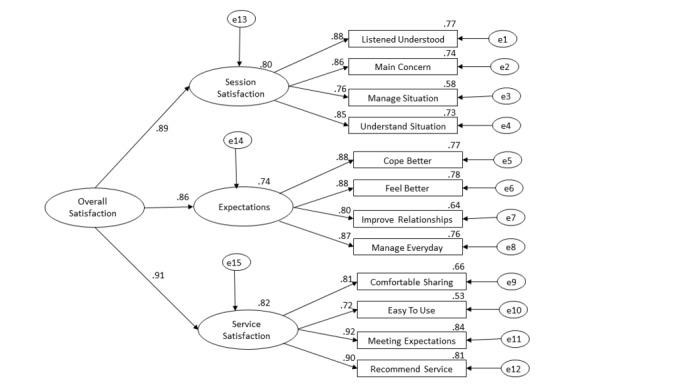
Modified three-factor model with total satisfaction as a second-order factor. Standardized coefficients (range .53-.92). e: error.

A CFA was run to further test the factor structure (see Table 1 and Figure 1). The following fit indexes were employed to assess model fit: chi-square closest to zero, root mean square error of approximation less than 0.06 (with the lower bound of its 90% confidence interval less than 0.05 to indicate close fit), the comparative fit index greater than 0.90, and adjusted goodness of fit index greater than 0.90 [39]. Based on the recommendation of Holmes-Smith et al [40], the model with the smallest Akaike information criterion (AIC) / consistent AIC (CAIC) was considered the best-fitting model.

Examination of the modification indexes revealed that the item “the session made me feel more hopeful/optimistic” may relate to both the session and expectations subscales. Only by deleting this item altogether did the model fit improve.

Models were rerun with the remaining 12 items. Results revealed that the original one-factor solution was not a good fit for the data. Consequently, the originally hypothesized three-factor solution was considered, along with other possibilities. Examination of the AIC and CAIC indexes showed that a three-factor model was the best fit to the data, but that a three-factor model with a higher order unidimensional construct of overall satisfaction was also a reasonable fit.

Note that the potential outcomes items included a “not applicable” option, as eheadspace clinicians felt that each of these outcomes may not be applicable for some clients. Consequently, to include participant responses to these items for the CFA (which cannot accept missing data), where only some items were not applicable, responses were substituted as neither agree nor disagree. Those who responded not applicable to all potential outcome items were excluded from analyses. Models were run with and without the substituted not applicable responses included, and all were a similar fit as shown in Table 1. Including those who chose not applicable to some questions, but still completed the full survey, reduces bias.

**Table 1:** Results of the confirmatory factor analysis.

Model	χ^2^ (*df*)	*P* value	RMSEA^a^	AGFI^b^	CFI^c^	AIC^d^	CAIC^e^
One factor	3418.7 (56)	<.001	0.180	0.591	0.821	3462.7	3606.3
Two factor	2008.7 (54)	<.001	0.140	0.723	0.896	2056.7	2213.3
Three factor without total	533.6 (51)	<.001	0.071	0.926	0.974	533.6	763.8
Three factor without N/A^f^ responses	880.9 (51)	<.001	0.094	0.884	0.949	934.9	1111.1
Final three-factor solution	787.8 (53)	<.001	0.086	0.897	0.961	837.8	1001.0

^a^RMSEA: root mean square error of approximation.

^b^AGFI: adjusted goodness of fit index.

^c^CFI: comparative fit index.

^d^AIC: Akaike information criterion.

^e^CAIC: consistent Akaike information criterion.

^f^N/A: not applicable.

### Reliability of Satisfaction Scale

Cronbach alpha statistics showed high internal consistency for the total scale and all subscales (see Table 2). If any individual items were removed the internal reliability of the scales would be reduced. Item-total correlations were calculated, and all subscales showed strong interitem correlations: session satisfaction items (*r=*.67-.82), potential outcomes items (*r=*.70-.80), and service satisfaction items (*r*=.66-.82). Furthermore, session, outcomes, and service items were moderately to strongly correlated (*r=*.34-.82). This further supports a second-order factor of total satisfaction.

Table 2 shows that overall young people were satisfied with eheadspace; all subscale means were above the scale midpoint and negatively skewed. Young people were most satisfied with the eheadspace service followed by their session, and they were least satisfied with their potential outcomes.

Client characteristics were not shown to influence total satisfaction scores (see Table 3). There were no significant differences in total satisfaction between males and females, adolescents and young adults, those who were heterosexual and those who were LGBTIQ, those who were an Aboriginal and/or Torres Strait Islander person and those who were not, and between young people who lived in metropolitan or regional locations. Those who attended because of a mental health issue were significantly less satisfied than those who attended for other reasons, although the effect was small. There was no effect of clinical stage.

Many service contact characteristics were significantly associated with satisfaction. Table 4 shows that young people who received esupport were more satisfied than those who received a brief service provision. Those who accessed prior to 11 pm were more satisfied than those who accessed late at night. Young people with contact of more than 30 minutes were more satisfied than those with shorter sessions. Clients who conveyed more than 500 words were more satisfied than those who conveyed 300 or less. Similarly, greater satisfaction was evident when clinicians conveyed more than 500 words. Young people who had not previously accessed a headspace center were more satisfied with eheadspace than those who had previously been to a headspace center. Wait time before session, previous eheadspace access, and referral source did not influence total satisfaction.

**Table 2:** Descriptive statistics for satisfaction scales (N=2280).

Satisfaction domain	Mean (SD)	Skewness	SE	Cronbach alpha
Session satisfaction	3.77 (1.07)	–1.085	0.051	.93
Potential outcomes	3.16 (0.86)	–0.604	0.051	.92
Service satisfaction	3.88 (0.91)	–0.320	0.051	.91
Total satisfaction	3.60 (0.83)	–0.918	0.051	.95

**Table 3:** Total satisfaction scores by client characteristics (N=2280).

Client characteristic		Satisfaction, mean (SD)	*F* (*df*1,*df*2)	*P* value	Partial η^2^
**Gender**			1.34 (2165,593)	.18	.028
	Male	3.66 (0.81)			
	Female	3.60 (0.83)			
**Age**			2.40 (2081,2278)	.02	.057
	12-17 years	3.64 (0.79)			
	18-25 years	3.56 (0.88)			
**Sexuality**			1.29 (2042,1054)	.20	.027
	Heterosexual	3.62 (0.84)			
	LGBTIQ^a^	3.57 (0.81)			
**Aboriginal and/or Torres Strait Islander person**			1.61 (2278,91)	.11	.032
	Yes	3.74 (0.85)			
	No	3.60 (0.83)			
**Area of residence**			–0.02 (2251,1262)	.98	.000
	Metro	3.61 (0.84)			
	Regional	3.61 (0.79)			
**Reason for attending**			–2.89 (1939,1208)	.004	–.062
	Mental health issue	3.67 (0.76)			
	Other	3.77 (0.72)			
**Clinical stage estimate^b^**			1.80 (3,956)	.15	.006
	Stage 0	3.72 (0.76)			
	Stage 1a	3.70 (0.70)			
	Stage 1b	3.61 (0.77)			
	Stage 2	3.51 (0.79)			

^a^LGBTIQ: Lesbian, Gay, Bisexual, Transgender, Intersex and Questioning.

^b^Note that no participants were rated in stages 3 or 4.

**Table 4:** Total satisfaction scores by service contact characteristics (N=2280).

Service characteristic		Satisfaction, mean (SD)	*F* (*df*1,*df*2)	*P* value	Partial η^2^
**Type of service**			11.40 (403,2278)	<.001	.282
	esupport	3.70 (0.75)			
	Brief service	3.04 (1.03)			
**Session start time**			17.87 (3,2276)	<.001	.023
	9 am-5 pm	3.66 (0.78)			
	5 pm-8 pm	3.66 (0.81)			
	8 pm-11 pm	3.66 (0.81)			
	11 pm-1 am	3.32 (0.94)			
**Wait time before session**			0.62 (3,2276)	.61	.001
	None	3.60 (0.87)			
	<5 minutes	3.63 (0.83)			
	<10 minutes	3.59 (0.80)			
	≥10 minutes	3.58 (0.83)			
**Session duration**			9.37 (3,1937)	<.001	.014
	<15 minutes	3.52 (0.88)			
	15-30 minutes	3.57 (0.82)			
	31-60 minutes	3.73 (0.72)			
	>60 minutes	3.84 (0.68)			
**Word count per client**			54.23 (3,2276)	<.001	.067
	1-300	3.46 (0.88)			
	301-500	3.69 (0.73)			
	>500	3.81 (0.71)			
**Word count per clinician**			73.09 (3,2276)	<.001	.088
	1-300	2.96 (1.06)			
	301-500	3.50 (0.87)			
	>500	3.73 (0.83)			
**Number of previous direct support sessions**			0.13 (3,2274)	.94	.000
	0	3.60 (0.85)			
	1	3.61 (0.80)			
	2	3.59 (0.85)			
	≥3	3.63 (0.75)			
**Referral information**			0.11 (1414,2234)	.92	.000
	Self-referred	3.60 (0.84)			
	Referred by other	3.60 (0.79)			
**Prior access to a headspace center**			–2.91 (790,1986)	.004	–.068
	Yes	3.50 (0.88)			
	No	3.63 (0.81)			

## Discussion

This study examined the implementation of a service satisfaction measure adapted from the headspace center-based satisfaction measure in the online youth mental health service, eheadspace. The aim was to determine the psychometric properties of the adapted measure in the online service environment and establish the level of service satisfaction among young people using the service, while examining whether this varied by client characteristics.

### Psychometric Properties of the Satisfaction Scale

The satisfaction measure showed good psychometric properties. There was high internal consistency for the overall satisfaction scale and its three subscales. A three-factor model of the eheadspace satisfaction feedback survey was shown to be the best fit to the data and the hypothesized subscales of session satisfaction, potential outcomes, and service satisfaction were evident. A three-factor model with a higher order unidimensional construct of overall satisfaction was also a reasonable fit. A higher order unidimensional overall satisfaction construct is supported by the literature. For example, Simmons et al [31] found support for a global construct of satisfaction in the development of a satisfaction scale for young people attending headspace centers.

Consistent with other service satisfaction research and literature, the measures of service satisfaction were negatively skewed, with most respondents indicating high satisfaction. The measure can help identify the characteristics of clients who are dissatisfied with the service, but it may be less useful in distinguishing among those with positive experiences [41,42]. Consequently, it may be difficult to demonstrate “improved satisfaction” when scores are already so high.

### Current Satisfaction of Young People Accessing eheadspace

Young people were generally very satisfied with eheadspace, and this did not vary across any demographic characteristics. No demographic differences were evident in this study unlike the findings of Rickwood et al [34] from face-to-face headspace center services, which found that men, younger clients, and those influenced by others to attend were less satisfied. This suggests that the eheadspace service is experienced in a similarly positive way across the demographics measured. However, the demographics of eheadspace clients are quite different from those of the headspace centers, as they are much more likely to be female and present with a high level of psychological distress [25]. This was reflected in that almost 78% of our participants were female, similar to the 80% who are female who access the service. Although the young men who accessed the service were equally satisfied, they were much less likely to access in the first place. The few client characteristics that were measured also did not substantially affect satisfaction. Those who attended because of a mental health issue were less satisfied than those who attended for other reasons, although the effect was small. It did not vary according to the estimate of clinical stage, which suggests that clients were equally highly satisfied regardless of the complexity of their issue. However, it should be noted that the measure of reason for attending was broad, comprising only mental health versus other issues, and the estimate of clinical stage was not obtained via a comprehensive clinical interview. It may be that satisfaction varies according to different types of mental health problems, as the online environment may be better suited to some than others and may be most useful as an adjunct to face-to-face therapy for some more serious and complex conditions.

Service contact characteristics were associated with satisfaction. Overall, young people who had a more comprehensive engagement with eheadspace were more satisfied. They were more satisfied when they received the more substantial esupport service rather than brief service provision, had a longer session, used more words, did not access the service very late at night, and had not previously experienced services from a face-to-face headspace center. This is consistent with research showing that eheadspace clients who had greater engagement showed more progress and depth of counseling and greater alleviation of psychological distress [43].

Similar to the findings regarding satisfaction from face-to-face headspace center services, satisfaction was lowest for perceived outcomes [34]. Many young people only access eheadspace once, so expectations of longer-term outcomes are unlikely to be well-formed. It is possible that many people might not know how to answer, especially if it is one of their first visits or their only visit, and if they only received information and not therapy as part of their session. Further, earlier research revealed that eheadspace clients have high service expectations [44], which are unlikely to be met with the minimal engagement that is typical.

### Limitations

The results of this study should be interpreted in light of its limitations. Notably, providing service satisfaction was optional, and the response rate was less than 10%. Although this was expected, it indicates likely response bias. It may be that only the more satisfied clients chose to complete the questionnaire. Finding ways to encourage more clients to complete the measure, and ensuring that those who are dissatisfied and who have less service engagement respond, are challenges for service satisfaction measures. Other indicators of service satisfaction should also be monitored, such as the number of site visits and drop-off rates.

Some of the measures were limited by the brief online nature of the data collection. In particular, the measure of clinical staging was an estimate. This measure was included to gain some understanding of the potential impact of client complexity, but it is acknowledged that a brief, online minimum data collection procedure cannot provide a robust measure of clinical stage such as can be ascertained through clinical interview within mental health services that adopt a transdiagnostic clinical staging approach (see [21]).

### Implications for Practice

The satisfaction results are regularly monitored and analyzed by the eheadspace service and evaluation staff and are used to guide service improvement. By identifying patterns in client satisfaction, the areas where the service needs to be tailored to better meet different client needs can be identified. Patterns over time can also be used to evaluate the impact of changes in practice.

Note that currently the satisfaction results are only analyzed in aggregate and not linked to individual clients. There is the potential to also examine session satisfaction monitored over time for individual clients through routine outcome monitoring. This is a repeated measurement process carried out therapeutically to improve treatment outcomes, which has been shown to improve treatment engagement and outcomes [45]. An even briefer measure may be required, however, to implement repeatedly, and clients would need to be informed that their individual feedback would be available to their service providers.

The satisfaction measure used in eheadspace solely assessed youth satisfaction as a form of feedback. Much youth mental health research has previously focused on parental and caregiver satisfaction [33]. Parents and caregivers often have different views about service satisfaction [41]. Their views are also important in assessing online youth mental health services, although this may be less relevant for online services where self-referral is predominant and young people can easily access without parental support. Nevertheless, parents and caregivers often play a primary role in whether younger people attend, engage with, and complete treatment, demonstrating the importance of their satisfaction [46]. Patel et al [1] suggested that both young person and parental feedback is essential in assessing satisfaction with mental health services and this should be explored in the online service domain.

Finally, the measure was adapted from the headspace center Youth Service Satisfaction Scale [34]. This scale has been successfully implemented in the face-to-face services, and it was of interest to have a comparative measure for the online service. Given the briefer and more transitory nature of the online service interactions, further adaptations may be required to make the measure most suitable to the online modality.

### Conclusions

This paper has described a service satisfaction measure for an online mental health service for young people. The eheadspace satisfaction measure is brief, and it demonstrated sound psychometric properties with good internal consistency and a clear factor structure. The measure showed high levels of satisfaction overall that were responsive to greater levels of engagement with the service. The measure could be adapted for use with other online youth mental health services, which are emerging as a critical setting for mental health service provision as part of stepped-care approaches in mental health [18].

## Abbreviations

AGFI: adjusted goodness of fit index
AIC: Akaike information criterion
CAIC: consistent Akaike information criterion
CFA: confirmatory factor analysis
CFI: comparative fit index
DSM-IV: Diagnostic and Statistical Manual of Mental Disorders Version 4
GAD: generalized anxiety disorder
LGBTIQ: Lesbian, Gay, Bisexual, Transgender, Intersex and Questioning
PCA: principal components analysis
RMSEA: root mean square error of approximation
